# Microglial activation state exerts a biphasic influence on brain endothelial cell proliferation by regulating the balance of TNF and TGF-β1

**DOI:** 10.1186/1742-2094-7-89

**Published:** 2010-12-06

**Authors:** Jennifer V Welser, Longxuan Li, Richard Milner

**Affiliations:** 1 Department of Molecular and Experimental Medicine, The Scripps Research Institute, 10550 North Torrey Pines Road, La Jolla, CA 92037, USA

## Abstract

**Background:**

Studies of cerebral ischemia and other neuroinflammatory states have demonstrated a strong association between new vessel formation and microglial recruitment and activation, raising the possibility that microglia may be involved in promoting angiogenesis. As endothelial cell proliferation is a fundamental early step in angiogenesis, the aim of this study was to test this hypothesis by examining the influence of microglial secreted factors on brain endothelial cell (BEC) proliferation using BrdU incorporation.

**Methods:**

Primary cultures of mouse BEC, microglia and astrocytes were used in this study. Proliferation of BEC was examined by BrdU incorporation. ELISA was used to quantify TNF and TGF-β1 levels within cell culture supernatants.

**Results:**

Microglia regulated BEC proliferation in a biphasic manner; microglia conditioned medium (MG-CM) from resting microglia inhibited, while that from activated microglia promoted BEC proliferation. A screen of microglial cytokines revealed that BEC proliferation was inhibited by TGF-β1, but promoted by TNF. ELISA showed that TNF and TGF-β1 were both present in MG-CM, and that while TGF-β1 dominated in resting MG-CM, TNF levels were massively increased in activated MG-CM, shifting the balance in favor of TNF. Antibody-blocking studies revealed that the influence of MG-CM to inhibit or promote BEC proliferation was largely attributable to the cytokines TGF-β1 and TNF, respectively.

**Conclusion:**

This data suggests that microglial activation state might be an important determinant of cerebral angiogenesis; inhibiting BEC proliferation and neovascularization in the normal central nervous system (CNS), but stimulating the growth of new capillaries under neuroinflammatory conditions.

## Background

Angiogenesis occurs in the central nervous system (CNS) not just during development [1], but also in pathological conditions, including cerebral ischemia [2], neoplasia [3], and neuroinflammation [4,5]. An improved understanding of the factors that control cerebral angiogenesis would be a big step forward in our attempts to regulate angiogenesis for therapeutic means, either to increase blood vessel growth during cerebral ischemia, or to inhibit vessel growth during neoplasia. Angiogenesis is regulated by a plethora of factors, including growth factors [6], cytokines [7], and extracellular matrix (ECM) molecules [8]. Within the CNS, it has been established that hypoxia promotes angiogenesis by at least two separate pathways. One involves hypoxia inducible factor-1α (HIF-1α)-dependent vascular endothelial growth factor (VEGF) release [9], and the other, that involves a HIF-1α-independent COX-2-dependent stimulation of PGE2, leading to angiopoietin-2 release [10]. In addition to soluble factors, ECM proteins also provide important instructional cues in angiogenesis [11], and recent work from our laboratory showing that fibronectin is strongly induced on angiogenic capillaries in the hypoxic CNS [12], as well as on angiogenic vessels in the developing CNS [13], suggests that this protein may also be important for cerebral angiogenesis.

In the normal adult CNS, brain endothelial cells (BEC) occupy an angiostatic state, and have the impermeable, tight-barrier characteristics of mature cerebral endothelium [14]. During cerebral ischemia and other neuroinflammatory conditions, vessels in the adult CNS mount an angiogenic response in which BEC proliferate to form new capillary sprouts [15,16]. Interestingly, studies of cerebral ischemic tissue have demonstrated a strong association between new vessel formation and microglial recruitment and activation [17,18], raising the possibility that microglia, the principal immune effector cells in the CNS, may actively promote angiogenesis. As endothelial cell proliferation is a fundamental early step in the angiogenic response, the aim of this study was to test this hypothesis by examining the influence of microglial secreted factors on BEC proliferation.

## Materials and methods

### Animals

The studies described have been reviewed and approved by The Scripps Research Institute Institutional Animal Care and Use Committee. All animals were maintained under pathogen-free conditions in the closed breeding colony of The Scripps Research Institute (TSRI).

### Cell Culture

Pure cultures of mouse brain endothelial cells (BEC) were prepared as previously described [19]. Briefly, brains were removed from 8 week-old C57Bl/6 mice, minced, dissociated for one hour in papain, centrifuged through 22% BSA to remove myelin, and then endothelial cells cultured in endothelial cell growth media (ECGM) consisting of Hams F12, supplemented with 10% FBS, Heparin, ascorbic acid, L-glutamine, penicillin/streptomycin (all from Sigma, St. Louis, MO) and endothelial cell growth supplement (ECGS) (Upstate Cell Signaling Solutions, Lake Placid, NY), on type I collagen (Sigma)-coated 6-well plates. Puromycin (4 μg/ml, Alexis GmbH, Grunberg, Germany) was included in culture media between days 1-3 to remove contaminating cell types. Endothelial cell purity was >99% as determined by CD31 in flow cytometry. For all experiments, BEC were used only for the first passage.

Mixed glial cultures were prepared from 0-2 day old C57Bl/6 mouse pups, as previously described [19], and maintained in poly-D-lysine coated T75 flasks in DMEM containing 10% fetal bovine serum (FBS) (all from Sigma). After 7-10 days, flasks were mechanically shaken to yield microglia, which were plated into uncoated 6 well plates. Microglial purity was >99% as determined by Mac-1 in flow cytometry. Pure astrocyte cultures were prepared as previously described [20], by plating neurospheres into poly-D-lysine coated 6-well plates and maintained in DMEM containing 10% FBS. Astrocyte purity of these cultures was >99% as determined by GFAP immunocytochemistry.

### Microglia-conditioned media (MG-CM)

Microglia were shaken off mixed glial cultures and plated in 6-well plates overnight. Media was then changed to serum-free DMEM containing N1-supplement, L-glutamine and penicillin/streptomycin (all from Sigma). Microglia were left unstimulated or activated with 1 μg/ml lipopolysaccharide (LPS, Sigma) to produce resting and activated MG-CM respectively. After 3 days, media was collected and filtered through a 0.22 μm filter before use in BEC studies. Astrocyte conditioned media (A-CM) was prepared in a similar manner.

### Brain endothelial cell proliferation assay

Glass coverslips were coated with collagen I (10 μg/ml, Sigma) for 2 hours, then washed, and BEC plated and cultured until cells reached ~50% confluence. For investigation of the effect of MG-CM, BEC were cultured in 67% ECGM and 33% MG-CM. Control cultures were maintained in 67% ECGM and 33% N1-supplemented media. In the function-blocking experiments, the anti-TNF and anti-TGF-β1 blocking antibodies and control antibodies (R&D, Minneapolis, MN) were used at 2 μg/ml. For the investigation of the influence of cytokines on BEC proliferation, BEC were cultured in ECGM with factors added across a concentration range, with the maximum indicated: IFN-α (10^3 ^U/ml, PBL Biomedical Labs), IFN-γ (10 U/ml, R&D), IL-6 (20 ng/ml, R&D), TNF (20 ng/ml, Genentech, San Francisco, CA), and TGF-β1 (20 ng/ml, R&D). BEC were cultured for 16 hours in the presence of BrdU (Invitrogen, Carlsbad, CA), then fixed in acid/alcohol and processed for BrdU immunocytochemistry according to the manufacturer's instructions. BEC proliferation was assessed by quantifying the number of BrdU-positive cells as a percentage of the total number of cells (Hoechst staining), and the results expressed as the mean ± SEM of four experiments. Statistical significance was assessed by using Student's t test, in which p < 0.05 was defined as statistically significant.

### ELISA analysis of glial cytokine production

Concentrations of TNF and TGF-β1 in microglial and astrocyte conditioned media were quantified by standard ELISA techniques using the Duoset ELISA system (R&D) according to the manufacturer's instructions. Results represent the mean ± SEM of 3 experiments, with each sample examined in triplicate within each experiment.

## Results

### Microglial secreted factors regulate BEC proliferation in a biphasic manner

**Figure 1:**
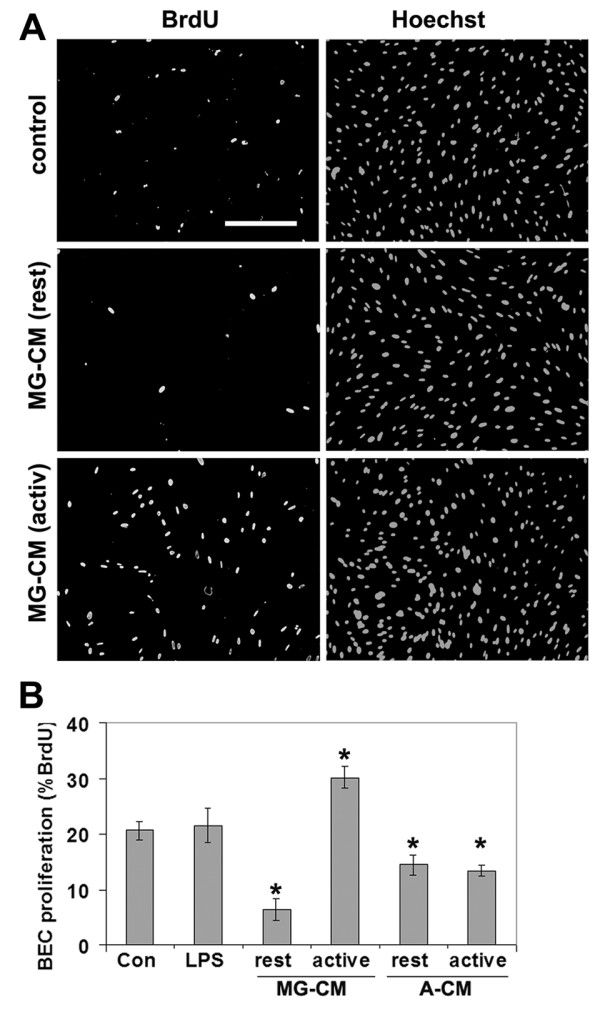
**Regulation of BEC proliferation by microglial-conditioned medium.** A. Dual immunocytochemistry for BrdU and Hoechst demonstrated that BEC proliferation was inhibited by conditioned media from resting microglia (MG-CM (rest)), but promoted by conditioned media from activated microglia (MG-CM (activ)). Scale bar = 100 μm. B. Quantification of BEC proliferation in response to microglia-conditioned media (MG-CM) and astrocyte-conditioned media (A-CM). BEC proliferation was examined over 16 hours, and expressed as the percentage of BEC that incorporated BrdU; all points represent the mean ± SEM of 4 experiments. MG-CM from resting or activated microglia, inhibited or promoted BEC proliferation, respectively. A-CM from resting and activated astrocytes both inhibited BEC proliferation. p < 0.05.

Microglia were purified from mixed glial cultures of postnatal mice, and grown in serum free media for 3 days, in the absence or presence of the activating agent lipopolysaccharide (LPS), to produce microglia-conditioned media (MG-CM) from resting (unstimulated) or activated microglia, respectively. The influence of resting or activated MG-CM on BEC proliferation was examined using BrdU incorporation. As shown in Figure 1, MG-CM had a clear biphasic influence on BEC proliferation rate. Compared to control conditions, resting MG-CM strongly inhibited BEC proliferation (6.4 ± 2.0% vs. 20.6 ± 1.6%, p < 0.001), while activated MG-CM had the opposite effect (30.1 ± 2.0% vs. 20.6 ± 1.6%, p < 0.002). The influence of astrocyte-conditioned media (A-CM) was also investigated. This revealed that resting and activated A-CM both inhibited BEC proliferation (resting A-CM 14.5 ± 1.8% vs. 20.6 ± 1.6%, p < 0.05, and activated A-CM 13.4 ± 1.0% vs. 20.6 ± 1.6%, p < 0.01).

### TNF and TGF-β1 have antagonistic effects on BEC proliferation

**Figure 2:**
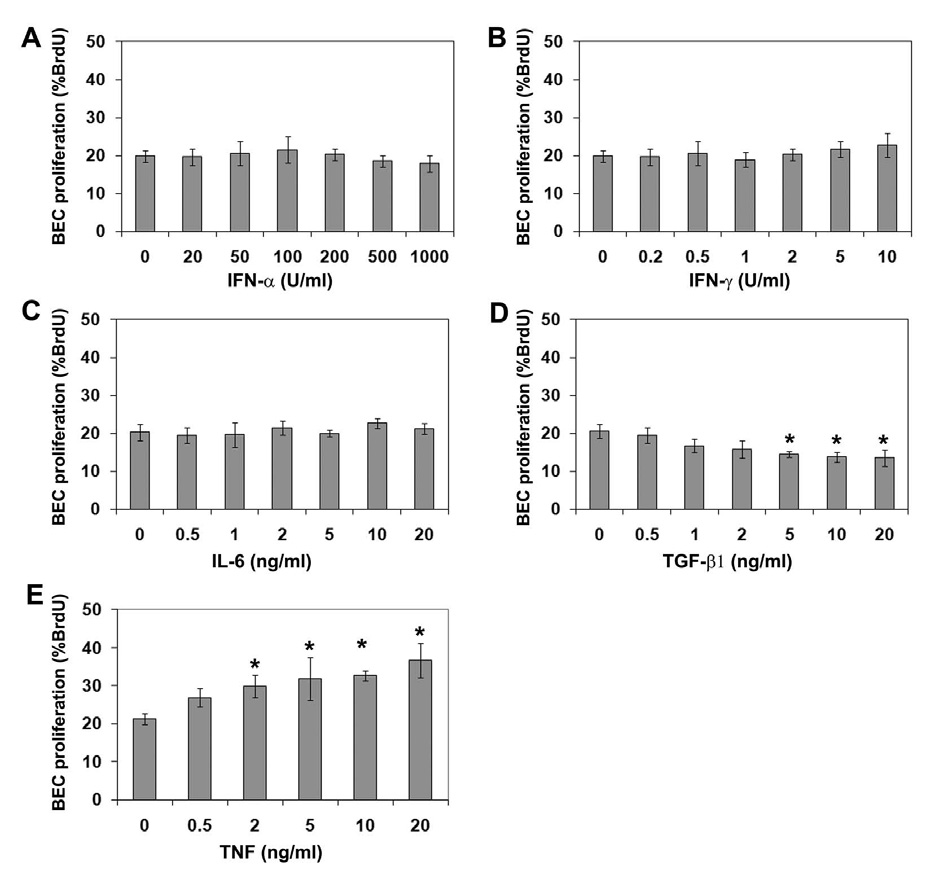
**Quantification of BEC proliferation in response to cytokines**. BEC proliferation was examined over 16 hours in the presence of different concentrations of the cytokines, IFN-α (A), IFN-γ (B), IL-6 (C), TGF-β1 (D), or TNF (E). BEC proliferation is expressed as the percentage of BEC that incorporated BrdU; all points represent the mean ± SEM of 4 experiments. Note that BEC proliferation was significantly stimulated by TNF, and inhibited by TGF-β1. p < 0.05.

Microglia produce a large number of cytokines, whose production is heavily-dependent on the state of microglial activation [21]. To identify which of these factors might be responsible for the microglial influence on BEC proliferation, we screened a panel of different cytokines for their ability to regulate BEC proliferation. This showed that TNF and TGF-β1 had the strongest, though opposing influences on BEC proliferation (Figure 2). TNF promoted BEC proliferation in a dose-dependent manner, which plateaued at a concentration of 10 ng/ml. Compared to control conditions (21.3 ± 1.4%), 10 ng/ml TNF increased the rate of BEC proliferation to 32.6 ± 4.5% (p < 0.01). In contrast, TGF-β1 reduced the rate of BEC proliferation, an effect which plateaued at a concentration of 10 ng/ml. Compared to control conditions, this concentration of TGF-β1 reduced BEC proliferation from 20.6 ± 1.9% to 13.8 ± 2.1% (p < 0.05). IFN-α, IFN-γ and IL-6 had no significant effect on BEC proliferation at any concentration tested.

### Microglia secrete TNF and TGF-β1, with the balance determined by microglial activation state

**Figure 3:**
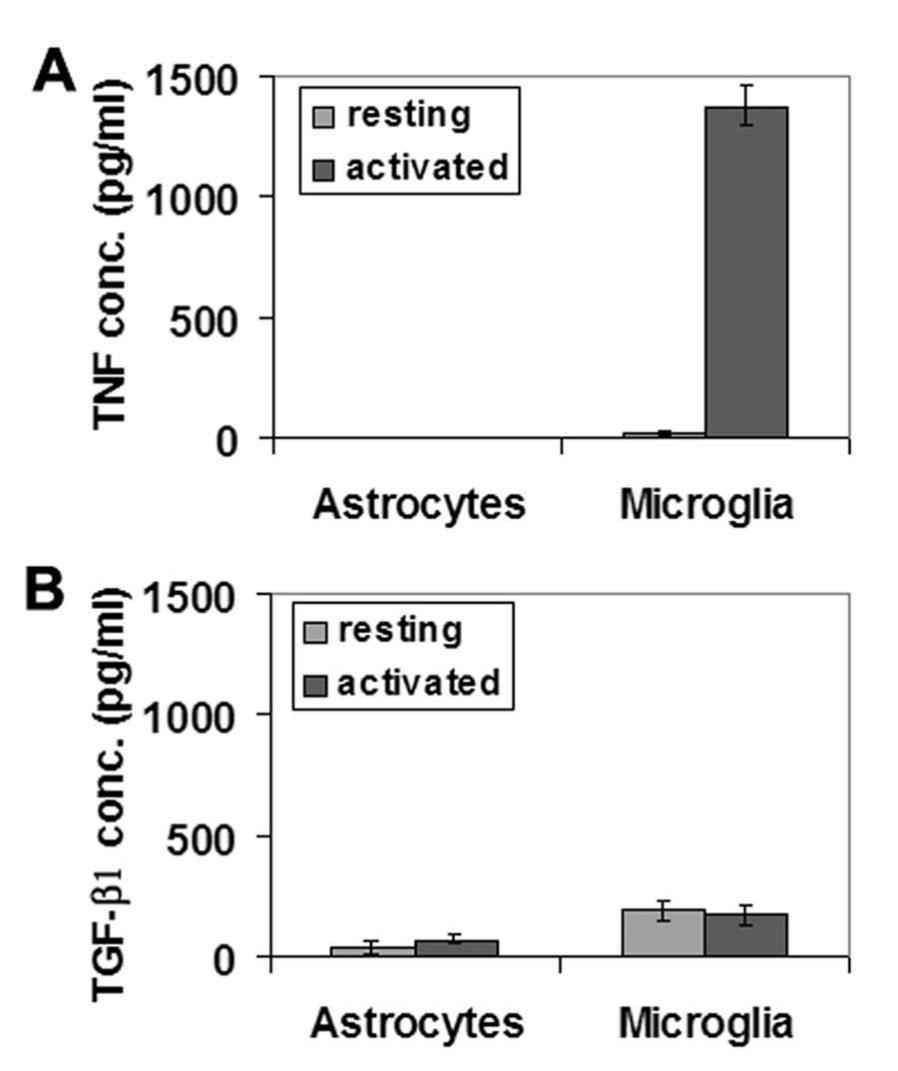
**ELISA quantification of TNF (A) and TGF-β1 (B) production by microglia or astrocytes, under resting or LPS-activated conditions**. All points are expressed in pg/ml concentration of cytokine and represent the mean ± SEM of 4 experiments. Microglial activation resulted in a massive increase in TNF production (50-fold), while astrocytes produced no TNF, even after LPS stimulation. Activation had no significant influence on TGF-β1 production by either cell type.

We have demonstrated that BEC proliferation rate is regulated by MG-CM in a biphasic manner; inhibited by resting MG-CM, and promoted by activated MG-CM. Furthermore, we have shown that BEC proliferation is inhibited by TGF-β1, but promoted by TNF. While it is known from previous studies that microglia can produce these two cytokines [22,23], we next used ELISA to investigate in our own cultures, whether MG-CM contained TNF or TGF-β1, and how this expression is regulated by LPS. As shown in Figure 3A, TNF was present in resting MG-CM (27.7 ± 8.6 pg/ml), but strongly increased (50-fold) in activated MG-CM (1381.2 ± 82.7 pg/ml). In contrast, TGF-β1 levels were not significantly different in resting (196.5 ± 42.3 pg/ml) or activated MG-CM (177.6 ± 43.4 pg/ml) (Figure 3B). A-CM contained no TNF, either before or after activation (Figure 3A). A-CM did contain TGF-β1, though this expression was not regulated by LPS treatment, and was at lower levels than MG-CM (39.2 ± 23.6 pg/ml and 75 ± 20.5 pg/ml in resting and activated A-CM, respectively). Thus, microglial activation switches the TNF/TGF-β1 balance heavily in favor of TNF.

### Microglial supernatants regulate BEC proliferation via TNF and TGF-β1

**Figure 4:**
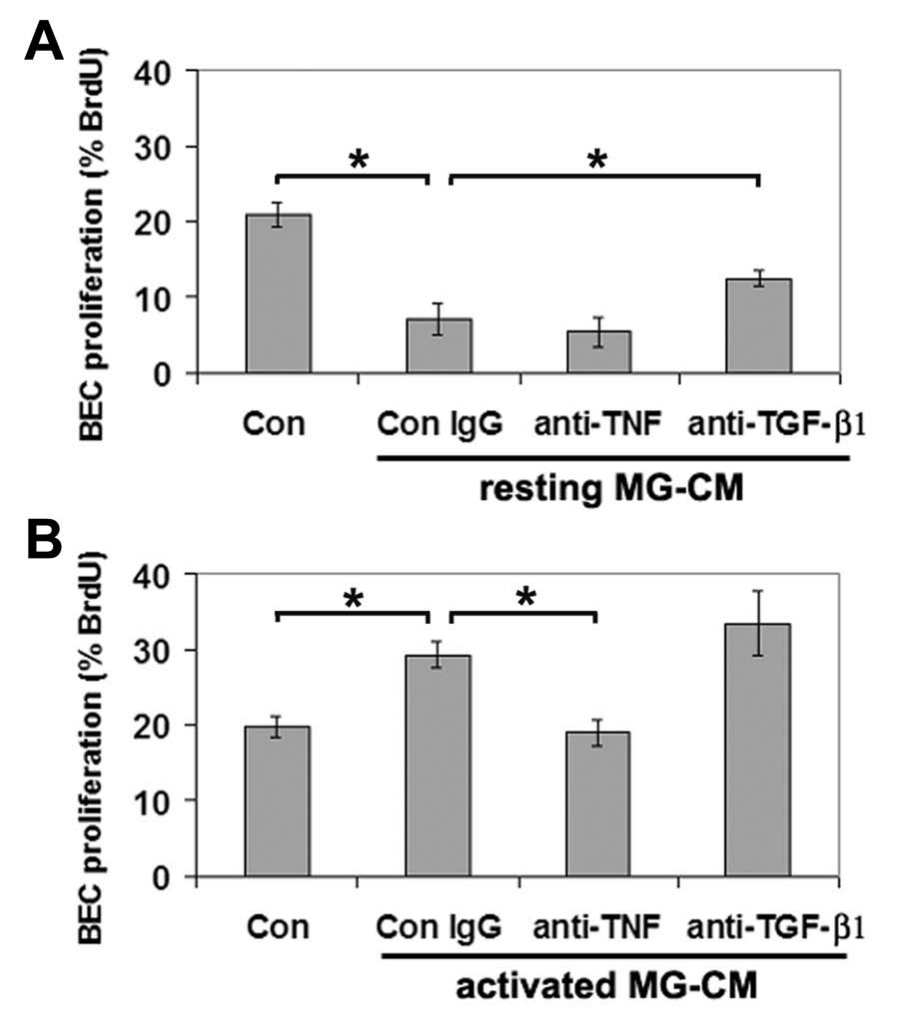
**Function-blocking experiments to examine the role of TNF and TGF-β1 in mediating the effects of MG-CM**. BEC proliferation is expressed as the percentage of BEC that incorporated BrdU; all points represent the mean ± SEM of 4 experiments. Note that the inhibitory influence of resting MG-CM was partially relieved by TGF-β1 antibodies (A) and conversely, the mitogenic influence of activated MG-CM was abrogated by anti-TNF antibodies (B). p < 0.05.

To address whether the influence of MG-CM on BEC proliferation is mediated by TNF or TGF-β1, we examined the influence of resting or activated MG-CM on BEC proliferation in the presence of function-blocking antibodies directed against TNF or TGF-β1. As shown in Figure 4A, compared to control conditions (20.9 ± 1.6%), resting MG-CM (Con IgG) significantly inhibited BEC proliferation (7.2 ± 2.0%, p < 0.005). This effect was significantly released by the anti-TGF-β1 antibody (12.5 ± 1.0%, p < 0.01). In contrast, compared to control conditions (19.7 ± 1.4%), activated MG-CM (Con IgG) significantly increased BEC proliferation (29.2 ± 1.7%, p < 0.01; Figure 4B), and this effect was significantly inhibited by the anti-TNF blocking antibody (18.9 ± 1.7%, p < 0.01). This data strongly suggests that TGF-β1 is in part responsible for the negative influence of resting MG-CM on BEC proliferation, and conversely, implicates TNF as a major factor responsible for the positive effect of activated MG-CM on BEC proliferation.

## Discussion

Cerebral angiogenesis occurs in the adult CNS in a number of conditions, including cerebral ischemia [2], neoplasia [3], and the neuroinflammatory conditions multiple sclerosis [5], and Alzheimer's disease [4]. In these conditions, angiogenesis is often associated with microglial accumulation and activation [17,18], raising the possibility that activated microglia may promote angiogenic events. In the current study, we directly examined this question in vitro, by exposing BEC to microglia-conditioned media. This revealed that microglia regulate BEC proliferation in a biphasic manner. Soluble factors from resting microglia inhibit, while those from activated microglia promote BEC proliferation, and these effects are largely attributable to the microglial cytokines TGF-β1 and TNF, respectively. This data suggests that in vivo, microglial activation state might be an important determinant of the earliest stage of cerebral angiogenesis, namely endothelial cell proliferation. This model predicts that in the normal CNS, tonic levels of TGF-β1 inhibit BEC proliferation, but that during cerebral ischemia or other neuroinflammatory processes, activated microglia ramp up TNF production, which promotes BEC proliferation.

### Microglial activation and angiogenesis

Microglia, as the primary immune effector cells in the CNS, play important roles in the surveillance and response to pathological insults. As the gatekeeper of the BBB, microglia are well positioned to mount a rapid aggressive response to noxious stimuli that enter the CNS. Under normal conditions, microglia occupy a resting "on surveillance" phenotype, but after stimulation, switch to an activated highly migratory, mitogenic phenotype, with upregulated production of inflammatory cytokines such as TNF and IFN-γ [21,24]. Within days of an ischemic insult, angiogenic remodeling can be observed at the ischemic penumbra [15,16], and interestingly, angiogenic vessels are often surrounded by inflammatory microglia and macrophages [17,18], suggesting that activated microglia and/or macrophages may be instrumental in promoting the angiogenic response to cerebral ischemia. While this idea has not yet been directly tested in the ischemic CNS, traumatic CNS injury leads to activation of microglia and macrophages, and drugs that block activation and proliferation of these cells, also inhibit neovascularization [25]. In addition, studies from outside the CNS support the concept that activated cells of the macrophage lineage directly promote angiogenic events. In tumor models, depletion of monocytes, the blood precursors of tissue macrophages, leads to marked reduction in tumor-associated angiogenesis [26], and conversely, addition of tissue macrophages strongly promotes neovascularization in corneal and rabbit ear chamber models [27,28]. Consistent with this, and in agreement with our own finding, conditioned media taken from activated macrophages directly promotes endothelial cell proliferation in vitro [29].

### The influence of TNF and TGF-β1 on angiogenic events

Our studies suggest that the positive and negative influences of microglia on BEC proliferation is mediated, at least in part, by the antagonistic cytokines TNF and TGF-β1, respectively. The influence of these cytokines on angiogenic events is still a matter of debate, with different studies demonstrating either pro- or anti-angiogenic effects, depending on the source of endothelial cells and the concentration of cytokine employed. TNF has been shown to both promote [30-32] and inhibit [33] angiogenic events, with one report demonstrating a negative effect of TNF on endothelial cell proliferation in vitro, but a stimulation of neovascularization in vivo [34], while another showed both positive and negative effects on endothelial cell proliferation, depending on the cellular source and cytokine combinations [35]. Similar apparently conflicting data have also been described for TGF-β1 [7,36]. In an attempt to reconcile these data, it has been suggested that the effects of these factors may have biphasic effects on angiogenesis, depending on cytokine concentration [36]. To directly investigate this possibility, we examined BEC proliferation across a wide range of cytokine concentrations, but in each case, this demonstrated a clear dose-response effect in one direction only, namely TNF promoting and TGF-β1 inhibiting BEC proliferation. Significantly, recent data has shown that the biphasic effect of TNF on endothelial cells can be explained by antagonistic functions of the TNF receptors TNFR1 and TNFR2, with TNFR2 stimulating endothelial cell survival, migration, and angiogenesis, while TNFR1 inhibits these functions [37,38]. In the current study, close examination of the TGF-β1 concentrations that mediated an inhibitory effect on BEC proliferation revealed an apparent difference in the potency of the endogenous form (present in resting MG-CM; 0.2 ng/ml) when compared with the pure recombinant form (5 ng/ml). Two reasons may explain this discrepancy. First, while the effect of recombinant TGF-β1 did not reach statistical significance until a concentration of 5 ng/ml, it did have an inhibitory trend at lower concentrations (0.5-1 ng/ml). Second and perhaps more important, in this study we used the human recombinant form of TGF-β1, and it is entirely plausible that the biological activity of this form on mouse BEC may not be as high as the endogenous murine form.

In summary, our data provides evidence that microglia, the principal immune effector cells in the CNS, can regulate BEC proliferation in a biphasic manner by altering the balance of TNF and TGF-β1. As BEC proliferation is an early stage of the angiogenic process, and new vessel formation leads to increased cerebral blood flow [39] and clinical outcome [40,41], our findings suggest that a controlled level of microglial activation and TNF release might prove beneficial in the treatment of stroke patients by promoting BEC proliferation and subsequent neovascularization. In light of the recent finding that microglia protect neurons from ischemia via a TNF-mediated mechanism [42], this approach has the potential to stimulate a positive outcome via two separate mechanisms.

## Conclusions

Our data demonstrate that microglia regulate BEC proliferation in a biphasic manner; microglia conditioned medium (MG-CM) from resting microglia inhibit, while that from activated microglia promote BEC proliferation. BEC proliferation was also inhibited by TGF-β1, but promoted by TNF. ELISA showed that TNF and TGF-β1 are both present in MG-CM, and that while TGF-β1 dominated in resting MG-CM, TNF levels were massively increased in activated MG-CM, shifting the balance in favor of TNF. Antibody-blocking studies revealed that the influence of MG-CM to inhibit or promote BEC proliferation was largely attributable to the cytokines TGF-β1 and TNF, respectively. Taken together, this data suggests that microglial activation state might be an important determinant of BEC proliferation, an early event in cerebral angiogenesis.

## Competing interests

The authors declare that they have no competing interests.

## Authors' contributions

JVW prepared the cell cultures, carried out the biochemical analysis and contributed to drafting the manuscript. LL participated in the design of the study and also assisted in manuscript preparation. RM conceived of the study, performed the proliferation studies, and helped to draft the manuscript. All authors read and approved the final manuscript.
